# Hematological abnormalities in clinically diagnosed non-alcoholic steatohepatitis: prevalence, clinical correlates, and fibrosis risk in a case–control study from Qatar

**DOI:** 10.3389/fmed.2026.1773499

**Published:** 2026-05-08

**Authors:** Aisha Al-Khinji, Dhafer Malouche, Noof Al-Thani, Azza Mustafa, Jazeel Abdulmajeed, Mohamed Ghaith Al-Kuwari

**Affiliations:** 1 College of Medicine, Qatar University, Doha, Qatar; 2 Translational Science Research Group, Health Cluster, Qatar University, Doha, Qatar; 3 Department of Mathematics and Statistics, College of Arts and Sciences, Qatar University, Doha, Qatar; 4 Primary Health Care Corporation, Doha, Qatar

**Keywords:** anemia, complete blood count, fatty liver disease, FIB-4, non-alcoholic steatohepatitis, Qatar, thrombocytopenia

## Abstract

**Background:**

Non-alcoholic steatohepatitis (NASH) is increasingly recognized as a systemic disease, yet the burden and determinants of hematological abnormalities in this population remain incompletely defined.

**Objectives:**

To compare the prevalence of complete blood count (CBC) abnormalities between patients with clinically diagnosed NASH and matched controls; to describe the demographic and clinical characteristics of NASH patients with anemia, neutropenia, or platelet abnormalities; and to examine the association between these abnormalities and non-invasive fibrosis scores.

**Methods:**

We conducted a case–control study using data from the Primary Health Care Corporation in Qatar. Adults with a clinical diagnosis of NASH based on ICD-10 coding (K75.81) in primary care records (*n* = 894) were matched 1:1 to controls without NASH on age and sex. CBC parameters were used to define anemia (sex-specific hemoglobin thresholds), neutropenia (absolute neutrophil count < 1.5 × 10^9^/L), thrombocytopenia (platelets < 150 × 10^9^/L), thrombocytosis (platelets > 400 × 10^9^/L), abnormal platelets (thrombocytopenia or thrombocytosis), and “any CBC abnormality.” Among NASH cases with available data (*n* = 765), we estimated prevalence, compared clinical characteristics, and fitted logistic regression models including age, sex, diabetes, hypertension, dyslipidemia, and the FIB-4 fibrosis score.

**Results:**

NASH patients had lower median white blood cell and platelet counts than controls and a higher prevalence of any CBC abnormality [35.8% vs. 28.4%; odds ratio (OR) 1.40, 95% confidence interval (CI) 1.12–1.75]. Thrombocytopenia was markedly more frequent in NASH (15.4% vs. 2.5%; OR 7.13, 95% CI 4.36–12.42). Within the NASH cohort, anemia (23.5%), neutropenia (5.4%) and abnormal platelets (18.6%) were more common in older patients and women, and in those with diabetes. Higher FIB-4 scores were associated with increased odds of anemia (OR 1.53 per 1-unit increase, 95% CI 1.21–1.99) and abnormal platelets (OR 3.60, 95% CI 2.31–6.09), and the prevalence of platelet abnormalities rose across FIB-4 risk categories.

**Conclusion:**

Hematological abnormalities are common in clinically diagnosed NASH and occur more frequently than in matched controls, with thrombocytopenia showing the strongest association and closely tracking non-invasive markers of advanced fibrosis. Routine CBC assessment may provide a simple tool to flag NASH patients at higher risk for clinically significant liver disease in primary care settings.

## Introduction

Metabolic dysfunction–associated steatotic liver disease (MASLD), previously known as non-alcoholic fatty liver disease (NAFLD), has emerged as the most common cause of chronic liver disease worldwide (1, 2). Contemporary meta-analyses suggest that steatotic liver disease affects roughly one third of the global population, with prevalence rising in parallel with obesity and type 2 diabetes (2). The Middle East and North Africa currently bear one of the highest burdens of MASLD: modeled estimates indicate that NAFLD/MASLD prevalence exceeds 40% in several countries, including Qatar (44.4%) (3). Local primary care data from Qatar further show that ultrasound-defined NAFLD is highly prevalent (approximately 58%) and strongly associated with metabolic syndrome and its components (4). Together, these data underscore MASLD as a major public health challenge in the region.

Initially considered a liver-limited condition, MASLD is now recognized as a multisystem disease with a broad range of extrahepatic complications. Large cohort studies and reviews have demonstrated strong associations between MASLD and cardiovascular disease, type 2 diabetes mellitus, chronic kidney disease, obstructive sleep apnea, and several malignancies (5, 6). Cardiovascular disease is the leading cause of death among patients with MASLD, exceeding liver-related mortality (6). These observations have shifted the clinical focus from isolated hepatic steatosis to a systemic disorder requiring integrated risk assessment across organ systems.

Hematologic abnormalities are increasingly recognized among the extrahepatic manifestations of MASLD but remain comparatively understudied. A recent review highlighted a spectrum of hematologic changes in NAFLD, including iron overload, dysmetabolic iron overload syndrome, and alterations in coagulation and platelet function, yet emphasized the paucity of high-quality data on cytopenias (7). In chronic liver disease more broadly, anemia, thrombocytopenia, and leukopenia may reflect portal hypertension with hypersplenism, chronic inflammation, nutritional deficiencies, bone-marrow suppression, or medication effects. However, most available evidence comes from patients with advanced cirrhosis or mixed-etiology liver disease, limiting our understanding of how often hematologic abnormalities occur in earlier stages of MASLD and whether they carry prognostic significance. Although MASLD has been linked to iron dysregulation and hemostatic changes, the present study focuses specifically on routinely available CBC abnormalities—namely hemoglobin concentration, neutrophil count, and platelet count—that can be readily assessed in primary care settings.

Platelet count and related indices occupy a central position at the interface between hematologic and hepatic disease. In NAFLD cohorts, lower platelet counts and altered platelet indices have been associated with more advanced fibrosis and cirrhosis, supporting their potential role as simple, non-invasive fibrosis markers (8). Time-dependent declines in platelet count, together with increases in the Fibrosis-4 (FIB-4) score, have also been linked to a higher risk of incident cirrhosis and liver-related events (9). These observations are clinically relevant because most widely used non-invasive fibrosis scores—such as FIB-4 and the aspartate aminotransferase to platelet ratio index (APRI)—combine age, transaminase levels, and platelet count to stratify the risk of advanced fibrosis (10–12). Multiple validation studies have shown that these simple scores achieve high negative predictive values for excluding advanced fibrosis in NAFLD and are increasingly recommended for use in primary care and general hepatology clinics (10–12).

Despite this, the prevalence and clinical correlates of predefined CBC abnormalities—particularly neutropenia, anemia, and thrombocytopenia—among individuals with clinically diagnosed NASH remain poorly characterized, and population-based or primary-care data are scarce. Most studies have either focused on a single CBC parameter (e.g., platelet count) as a surrogate of fibrosis or have been restricted to hospital-based or biopsy-proven cohorts. Systematic descriptions of the full spectrum of CBC abnormalities in population-based or primary-care–linked MASLD cohorts, and how these abnormalities relate to metabolic risk factors and non-invasive fibrosis scores, are lacking (7, 8). This gap is especially relevant in high-burden regions such as the Middle East, where laboratory-based tools that can be embedded into routine primary care workflows are needed to prioritize patients for further liver assessment.

Qatar provides a pertinent setting in which to address these questions. The country has a high prevalence of obesity, type 2 diabetes, and MASLD (3, 4), alongside a rapidly developing primary health care system. The Primary Health Care Corporation (PHCC) maintains comprehensive electronic health records that include detailed laboratory measurements, such as CBC and liver biochemistry, and thus represents an ideal platform for characterizing hematologic manifestations of fatty liver disease at the population level.

Within this context, the present study aims (i) to estimate the prevalence of predefined CBC abnormalities among adults with clinically diagnosed NASH in a primary care population in Qatar; (ii) to describe the demographic and clinical characteristics of participants with coexisting NASH and anemia, neutropenia, or abnormal platelet counts; and (iii) to explore the association between these hematologic abnormalities and non-invasive fibrosis scores (FIB-4 and APRI). By leveraging routinely available blood tests, this work seeks to clarify how hematologic abnormalities fit within the broader MASLD phenotype and to inform risk stratification strategies that are feasible in primary health care settings.

## Methods

### Study design and data sources

We conducted an observational case–control study using data from the Primary Health Care Corporation (PHCC), the main provider of primary health care services in Qatar. PHCC maintains comprehensive electronic medical records that include demographic information, clinical diagnoses, laboratory measurements, and imaging data for registered patients. For the present analysis, we used an analytical dataset in which PHCC patients were classified as having or not having a diagnosis of non-alcoholic steatohepatitis (NASH) based on clinical records.

### Case definition and inclusion/exclusion criteria

*Cases* were adults ( ≥ 18 years) registered at PHCC with a recorded clinical diagnosis of NASH, identified using the International Classification of Diseases, Tenth Revision (ICD-10) code K75.81 (non-alcoholic steatohepatitis) or related fatty liver disease codes (K76.0 and K76.89) assigned by primary care physicians. In routine PHCC clinical practice, these diagnoses were typically supported by: (1) hepatic ultrasound demonstrating steatosis (echogenicity consistent with fatty infiltration), (2) elevated liver enzymes (ALT and/or AST), and (3) clinical exclusion of significant alcohol consumption (defined as > 21 units/week in men or > 14 units/week in women), viral hepatitis, autoimmune liver disease, and other known causes of hepatic steatosis. Histological confirmation was not available in this primary care setting, which reflects real-world diagnostic practice and is acknowledged as a limitation.

*Controls* were adults without any recorded diagnosis of NASH, NAFLD, or related liver disease codes (K75.81, K76.0, K76.89) in their PHCC medical records, frequency-matched 1:1 to cases on age (5-year strata) and sex. Controls were drawn from the same underlying PHCC-registered patient population as cases, ensuring similar access to care. Controls were not required to be free of metabolic comorbidities (such as diabetes, hypertension, or dyslipidemia), as these conditions are intrinsically associated with NASH and excluding them would introduce healthy-volunteer bias; instead, all such comorbidities were explicitly adjusted for in regression analyses.

*Exclusion criteria* (applied to both cases and controls): age < 18 years; diagnosis codes indicating alcoholic liver disease (K70.x), viral hepatitis (B15–B19), autoimmune hepatitis (K75.4), or primary biliary/sclerosing cholangitis (K74.3, K83.0); and absence of any laboratory data in the PHCC record.

The sample size of 894 cases was determined by the availability of eligible patients with a NASH diagnosis in the PHCC electronic health record system during the study period. No formal a priori power calculation was conducted; however, this sample size provides > 80% power to detect an odds ratio of ≥ 1.5 for the primary outcome (any CBC abnormality) at a two-sided α = 0.05, assuming a baseline prevalence of approximately 28% in controls.

Hematological parameters were measured by automated hematology analyzers operating within PHCC’s ISO 15189-accredited central and satellite laboratories. All analyzers were subject to internal quality control procedures and external quality assurance schemes according to PHCC laboratory standards. Specific analyzer models were not uniformly recorded in the electronic health record extract available for this study.

### Study population

The study population comprised 1,788 adults: 894 NASH cases and 894 controls without NASH. Cases and controls were selected from the same underlying PHCC patient population and were frequency-matched in a 1:1 ratio on age (5-year strata) and sex to minimize confounding by these variables. Baseline characteristics of the two groups are presented in Table 1. Complete blood count (CBC) data were available for 765/894 (85.6%) NASH cases and 682/894 (76.3%) controls, and analyses involving CBC parameters were restricted to participants with available measurements.

**TABLE 1:** Baseline characteristics of NASH cases and matched controls.

Characteristic	Control (*n* = 894)	NASH (*n* = 894)	*P*-value
N	894	894	–
Age, years, mean (SD)	50.3 (13.2)	50.3 (13.2)	0.956
Female, n (%)	382 (42.7)	382 (42.7)	1.000
Diabetes mellitus, n (%)	553 (61.9)	556 (62.2)	0.922
Hypertension, n (%)	407 (45.5)	465 (52.0)	0.007
Dyslipidemia, n (%)	420 (47.0)	501 (56.0)	< 0.001

### Outcome definitions

CBC parameters were obtained from routine laboratory measurements in PHCC records at or near the time of study enrolment. When more than one measurement was available, we preferentially used the most recent value and, if missing, reverted to the earlier value. We defined hematological outcomes using standard clinical thresholds:

- *Anemia:* hemoglobin (Hgb) < 13 g/dL in men or < 12 g/dL in women, based on World Health Organization criteria.
- *Absolute neutrophil count (ANC):* calculated as white blood cell (WBC) count × neutrophil percentage / 100, expressed in × 10^9^/L.
- *Neutropenia:* ANC < 1.5 × 10^9^/L.
- *Thrombocytopenia:* platelet count < 150 × 10^9^/L.
- *Thrombocytosis:* platelet count > 400 × 10^9^/L.
- *Abnormal platelets:* presence of thrombocytopenia or thrombocytosis.
- *Any CBC abnormality:* presence of anemia, neutropenia, or abnormal platelets.

These outcomes were evaluated both in the full case–control sample (for Aim 1) and within the NASH cohort only (for Aim 2 and Aim 3).

### Non-invasive fibrosis scores

To characterize liver disease severity among NASH cases, we calculated two non-invasive fibrosis scores using routinely measured aspartate aminotransferase (AST), alanine aminotransferase (ALT), and platelet count values:

- *Fibrosis-4 (FIB-4) index*:
$$\mathrm{FIB}-4=\frac{\mathrm{Age}\,\left(\mathrm{years}\right)\times \mathrm{AST}\,\left(U/L\right)}{\mathrm{Platelet}\,\mathrm{count}\,\left(10^{9}/L\right)\times \sqrt{\mathrm{ALT}\,\left(U/L\right)}}$$
  FIB-4 values were categorized into three risk groups commonly used in clinical practice: low risk of advanced fibrosis (<1.30), indeterminate risk (1.30–2.67), and high risk ( > 2.67) (10, 12).
- *AST to Platelet Ratio Index (APRI):*
$$\mathrm{APRI}=\frac{\mathrm{AST}\,\left(U/L\right)/40}{\mathrm{Platelet}\,\mathrm{count}\,\left(10^{9}/L\right)}\times 100$$
  assuming an upper limit of normal (ULN) for AST of 40 U/L. APRI was similarly categorized as low risk ( < 0.5), intermediate risk (0.5–1.5), and high risk ( > 1.5) for significant fibrosis (12).

Because AST, ALT, and platelet values were not available for all NASH participants, FIB-4 and APRI were calculated in the subset with complete data; this subset was used in analyses that incorporated fibrosis scores.

### Covariates

Demographic and clinical variables were derived from PHCC records. Age (years) was treated as a continuous variable. Sex was coded as male or female. The presence of type 2 diabetes mellitus, hypertension, and dyslipidemia was ascertained from clinician-recorded diagnoses in the primary care data and coded as binary variables (yes/no). These comorbidities were selected a priori because of their strong association with fatty liver disease and potential influence on hematologic parameters.

### Missing data

We first described the extent of missing data for key laboratory variables among NASH cases. In this cohort, CBC parameters (hemoglobin, WBC, and platelet count) were each available for 765/894 participants (14.4% missing), whereas AST and ALT values, and therefore FIB-4 and APRI scores, were missing in approximately 62% of participants (Supplementary Table 1). To assess whether missingness in liver enzyme data introduced selection bias, we compared characteristics of NASH patients with versus without available FIB-4 data (Supplementary Table 8). These comparisons inform the interpretation of fibrosis-related analyses. Analyses were performed using a complete-case approach for each outcome: participants with missing data for a given outcome or covariate were excluded from the corresponding analysis but retained for analyses of other outcomes.

### Statistical analysis

For Aim 1, we compared baseline characteristics and CBC parameters between NASH cases and controls. Continuous variables were summarized as means and standard deviations (SD) or medians and interquartile ranges (IQR), depending on their distribution, and compared using two-sample *t* tests or Wilcoxon rank-sum tests as appropriate. Categorical variables were summarized as frequencies and percentages and compared using chi-square tests. We estimated the prevalence of each CBC abnormality in cases and controls with corresponding 95% confidence intervals (CIs) and calculated odds ratios (ORs) for NASH versus control status.

For Aim 2, within the NASH cohort we estimated the prevalence of anemia, neutropenia, thrombocytopenia, thrombocytosis, abnormal platelets, and any CBC abnormality. We compared demographic and clinical characteristics between NASH patients with and without each abnormality using the same univariable methods described above. We also described the distribution of CBC parameters and fibrosis scores graphically.

For Aim 3, we fitted multivariable logistic regression models to identify factors independently associated with each hematologic outcome (anemia, neutropenia, and abnormal platelets) among NASH patients. A base model (Model 1) included age, sex, diabetes, hypertension, and dyslipidemia as predictors. An extended model (Model 2) further included the FIB-4 score as a continuous variable to examine the relationship between non-invasive fibrosis and CBC abnormalities. Results are reported as adjusted odds ratios (aORs) with 95% CIs.

#### Important methodological consideration

Because FIB-4 incorporates both age and platelet count in its formula, there is inherent mathematical coupling between this score and the outcomes of interest (particularly abnormal platelets). The strong associations observed between FIB-4 and platelet abnormalities in Model 2 should therefore be interpreted as partially reflecting this mathematical relationship rather than purely independent biological associations. Despite this limitation, we included FIB-4 in Model 2 because of its widespread clinical use and to allow comparison with prior literature.

For each outcome, we compared the relative fit of the base and extended models using the Akaike information criterion (AIC); lower AIC values were interpreted as indicating better model fit, acknowledging that the models were estimated on different sample sizes because FIB-4 was available only in a subset of participants. As a sensitivity analysis, we repeated the extended models replacing FIB-4 with APRI. Since APRI incorporates AST and platelet count but not age, it has less mathematical coupling with platelet outcomes than FIB-4, providing a partial check on the directionality of associations (Supplementary Table 9). Statistical significance was defined as a two-sided *p*-value < 0.05, without formal adjustment for multiple comparisons given the exploratory nature of the analyses.

All analyses were conducted using R software (version 4.5.1; R Foundation for Statistical Computing, Vienna, Austria).

### Ethical considerations

The study was conducted in accordance with the principles of the Declaration of Helsinki. The present analysis of de-identified data from PHCC was approved by the relevant institutional review boards in Qatar. No additional contact with participants was required.

## Results

### Study population and data completeness

A total of 1,788 participants were included: 894 with clinically diagnosed NASH and 894 matched controls without NASH. By design, age and sex distributions were identical between groups (mean age 50.3 years in both; 42.7% female), while the prevalence of diabetes was similarly high in cases and controls (62.2% vs. 61.9%; Table 1). NASH cases more frequently had hypertension (52.0% vs. 45.5%; *p* = 0.007) and dyslipidemia (56.0% vs. 47.0%; *p* < 0.001).

Among NASH participants, CBC parameters (hemoglobin, WBC, platelets) were available for 765/894 (85.6%), whereas AST/ALT and derived non-invasive fibrosis scores (FIB-4 and APRI) were only available in approximately 38% (Supplementary Table 1). Analyses were therefore performed on a complete-case basis for each outcome.

### Aim 1: case–control comparison of CBC abnormalities

Median hemoglobin concentrations were similar in NASH cases and controls (13.7 vs. 13.5 g/dL; *p* = 0.11), but NASH patients had lower WBC counts (6.4 vs. 7.0 × 10^9^/L; *p* < 0.001), lower absolute neutrophil counts (3.30 vs. 3.65 × 10^9^/L; *p* < 0.001) and lower platelet counts (235 vs. 261 × 10^9^/L; *p* < 0.001; Table 2). Lymphocyte percentages did not differ materially between groups.

**TABLE 2:** CBC parameters in NASH cases vs. controls [median (IQR)].

Parameter	Control	NASH	*P*-value
N with CBC data	682	765	–
Hemoglobin, g/dL	13.5 (12.3–14.8)	13.7 (12.3–15.0)	0.111
WBC, × 10^9^/L	7.0 (5.7–8.2)	6.4 (5.2–8.0)	< 0.001
ANC, × 10^9^/L	3.7 (2.8–4.8)	3.3 (2.5–4.3)	< 0.001
Platelets, × 10^9^/L	261.0 (218.0–301.0)	235.0 (185.0–282.0)	< 0.001

Values are median (IQR) unless otherwise specified.

**FIGURE 1:**
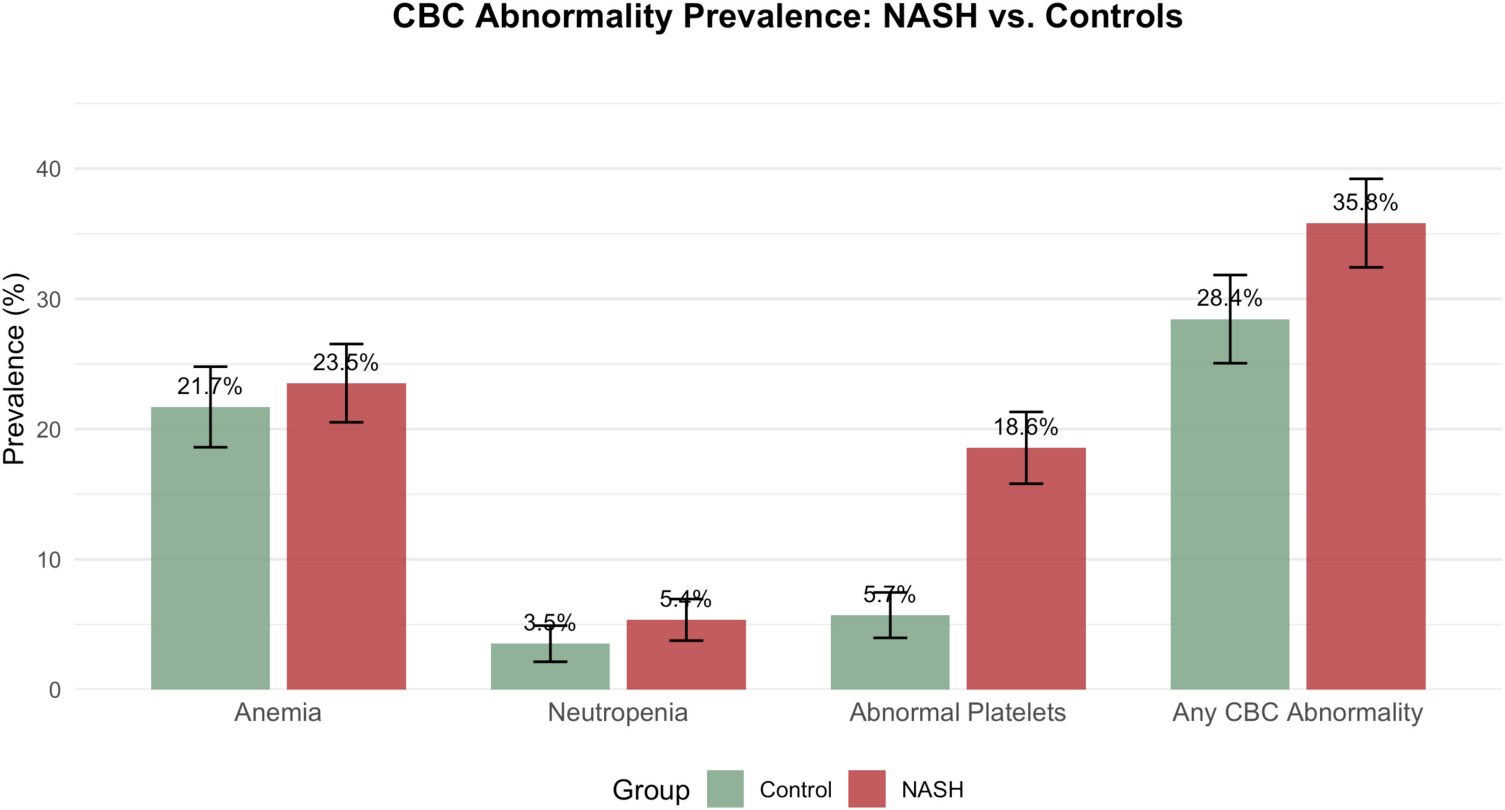
Prevalence of CBC abnormalities: NASH cases vs. matched controls.

The prevalence of at least one CBC abnormality was higher in NASH cases than in controls (35.8% vs. 28.4%), corresponding to an odds ratio (OR) of 1.40 (95% confidence interval [CI] 1.12–1.75; Figure 1 and Table 3). This difference was driven predominantly by platelet abnormalities. Thrombocytopenia affected 15.4% of NASH patients compared with 2.5% of controls (OR 7.13, 95% CI 4.36–12.42), and any platelet abnormality (thrombocytopenia or thrombocytosis) occurred in 18.6% vs. 5.7% (OR 3.76, 95% CI 2.62–5.51). Anemia (23.5% vs. 21.7%; OR 1.11, 95% CI 0.87–1.42) and neutropenia (5.4% vs. 3.5%; OR 1.55, 95% CI 0.93–2.63) were modestly more frequent in NASH but did not reach conventional statistical significance.

**TABLE 3:** Prevalence of CBC abnormalities: NASH vs. controls.

Outcome	Control, % (95% CI)	NASH, % (95% CI)	OR (95% CI)
Anemia	21.7 (18.6–24.8)	23.5 (20.5–26.5)	1.11 (0.87–1.42)
Neutropenia	3.5 (2.1–4.9)	5.4 (3.8–7.0)	1.55 (0.93–2.63)
Thrombocytopenia	2.5 (1.3–3.7)	15.4 (12.9–18.0)	7.13 (4.36–12.42)
Thrombocytosis	3.2 (1.9–4.6)	3.1 (1.9–4.4)	0.97 (0.54–1.76)
Any platelet abnormality	5.7 (4.0–7.5)	18.6 (15.8–21.3)	3.76 (2.62–5.51)
Any CBC abnormality	28.4 (25.1–31.8)	35.8 (32.4–39.2)	1.40 (1.12–1.75)

OR, Odds ratio for NASH vs. Control (reference).

### Aim 2: prevalence and characteristics of CBC abnormalities in NASH

**FIGURE 2:**
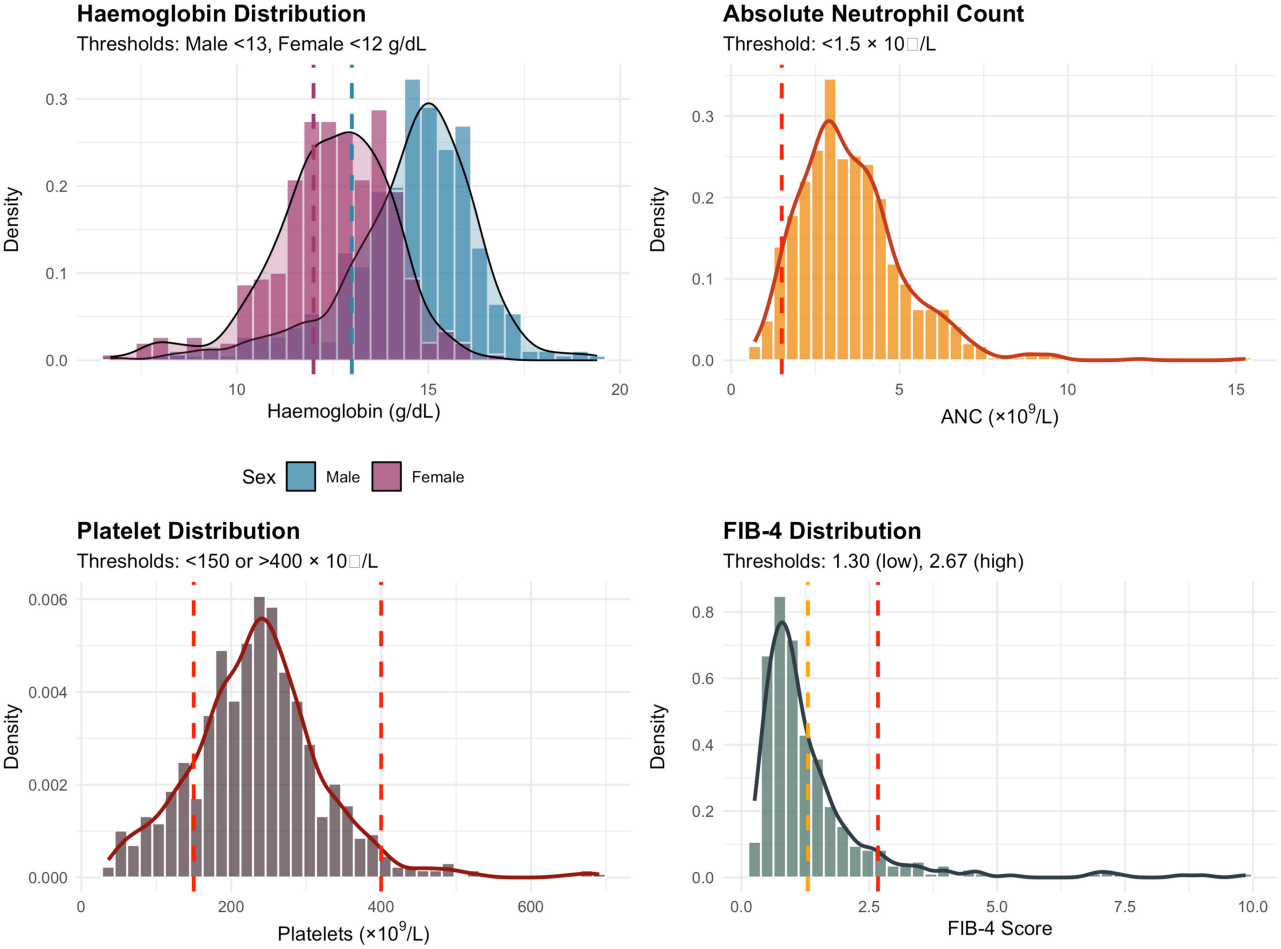
Distribution of CBC parameters in NASH patients. Dashed lines indicate clinical thresholds for abnormality.

Within the NASH cohort with available CBC data (*n* = 765), anemia was present in 23.5%, neutropenia in 5.4%, thrombocytopenia in 15.4%, and thrombocytosis in 3.1%; overall, 18.6% had any platelet abnormality and 35.8% had at least one of the predefined CBC abnormalities (Table 4). Distributions of hemoglobin, ANC, platelet count and FIB-4, together with the corresponding clinical thresholds, are shown in Figure 2.

**TABLE 4:** Prevalence of CBC abnormalities within the NASH cohort.

Outcome	n/N	Prevalence (%)
Anemia	180/765	23.5
Neutropenia	41/765	5.4
Thrombocytopenia	118/765	15.4
Thrombocytosis	24/765	3.1
Any platelet abnormality	142/765	18.6
Any CBC abnormality	274/765	35.8

NASH patients with anemia were older (mean 54.2 vs. 49.2 years; *p* < 0.001) and more often female (64.4% vs. 38.5%; *p* < 0.001) than those without anemia, and had a higher prevalence of diabetes (73.9% vs. 59.3%; *p* = 0.001) (Supplementary Table 2). Mean FIB-4 and APRI values were also higher among anemic patients (FIB-4 2.08 vs. 1.21; APRI 0.65 vs. 0.48; both *p* ≤ 0.002).

Similar patterns were observed for platelet abnormalities. Patients with abnormal platelet counts were on average almost 9 years older than those with normal platelets (57.8 vs. 48.7 years; *p* < 0.001) and were more frequently female (61.3% vs. 40.8%; *p* < 0.001), with higher prevalences of diabetes (83.1% vs. 58.1%; *p* < 0.001) and hypertension (66.2% vs. 48.6%; *p* < 0.001) (Supplementary Table 3). Mean FIB-4 and APRI values were substantially greater in those with platelet abnormalities (FIB-4 3.18 vs. 1.12; APRI 0.90 vs. 0.45; both *p* < 0.001).

**FIGURE 3:**
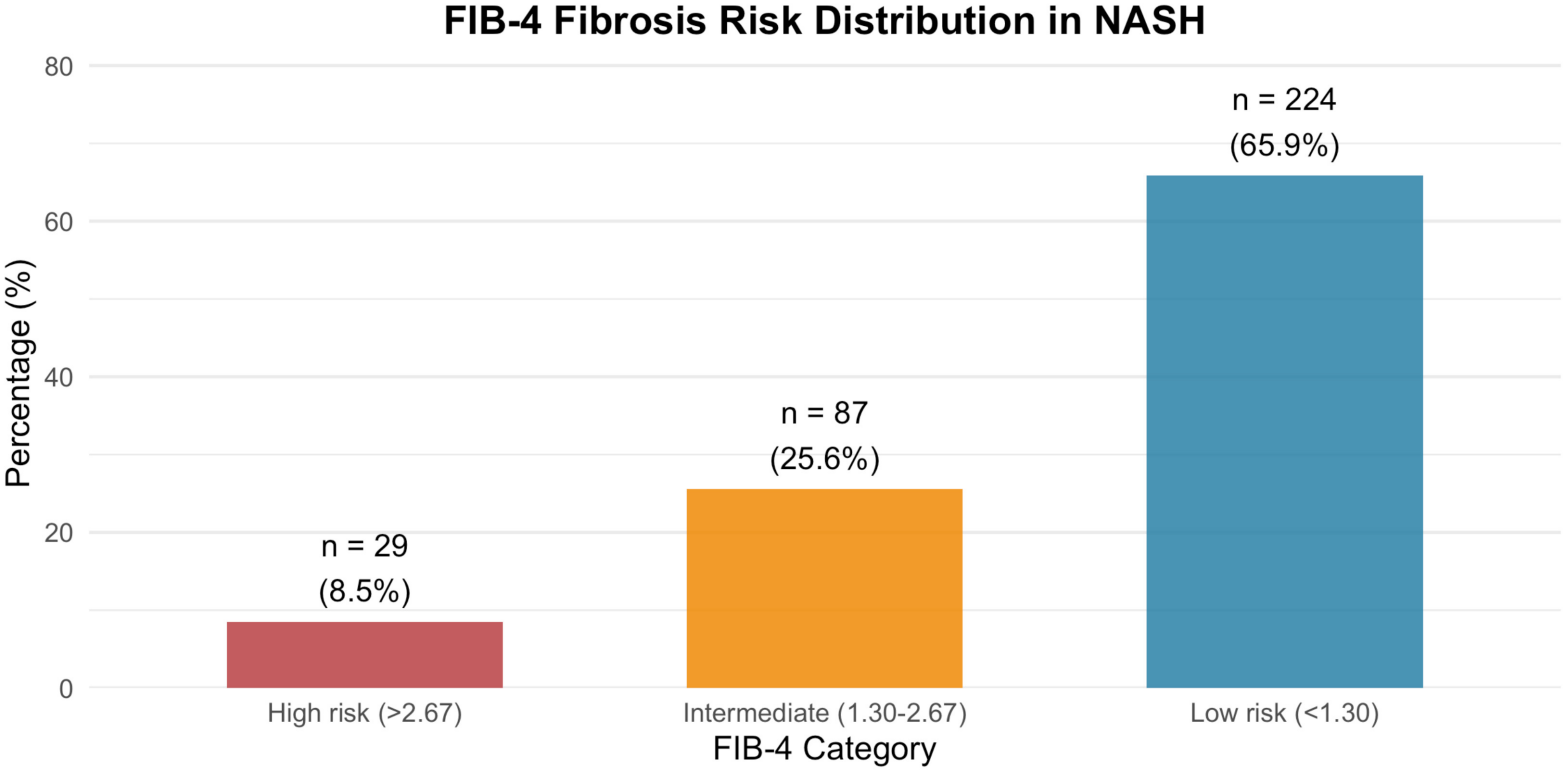
Distribution of FIB-4 risk categories in NASH patients.

**FIGURE 4:**
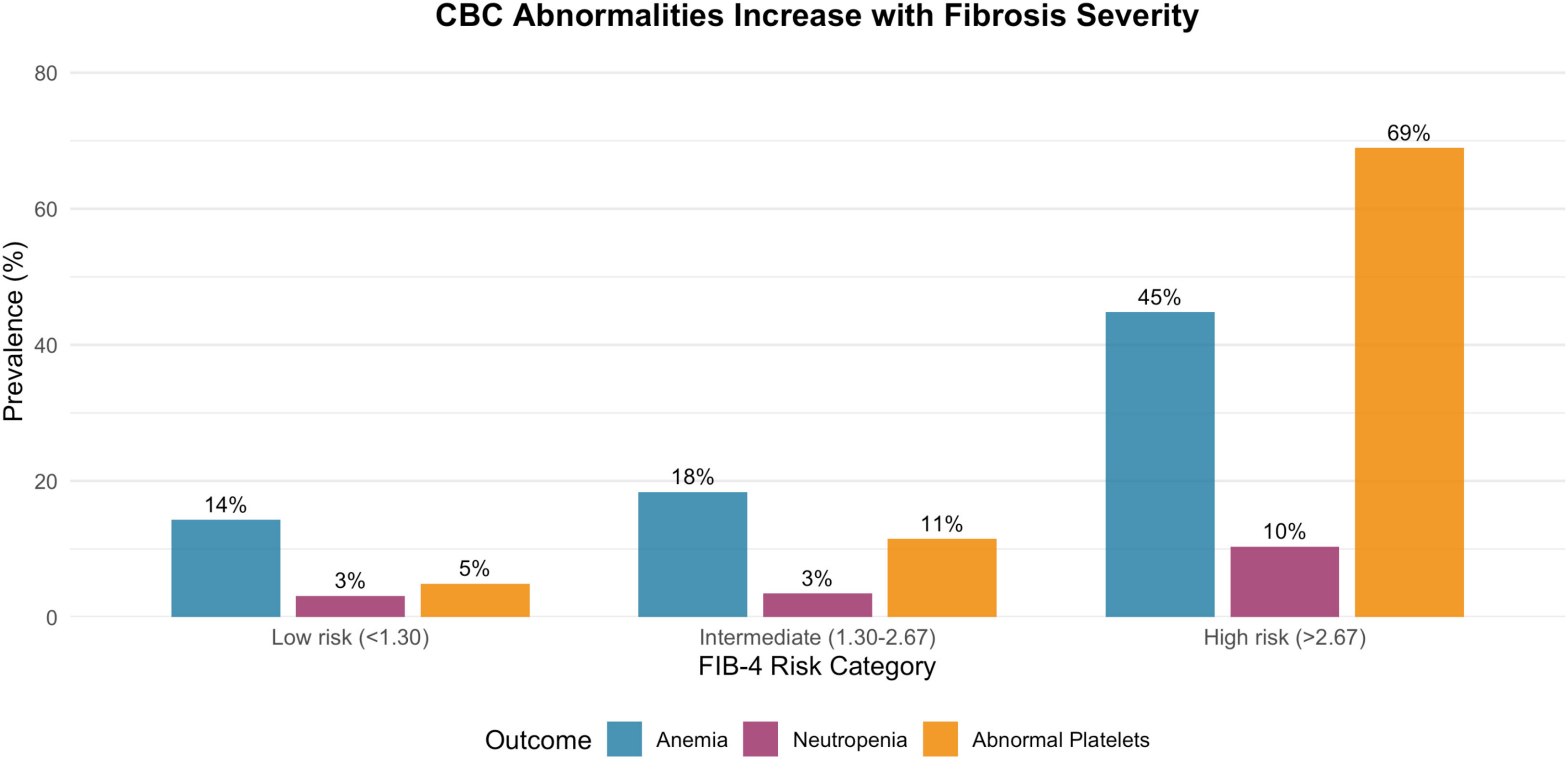
Prevalence of CBC abnormalities by FIB-4 fibrosis risk category.

Among the 340 NASH patients with available data to calculate FIB-4, 65.9% were classified as low risk of advanced fibrosis (FIB-4 < 1.30), 25.6% as indeterminate (1.30–2.67), and 8.5% as high risk (>2.67; Figure 3). The prevalence of hematologic abnormalities increased stepwise across these fibrosis categories (Figure 4). For example, abnormal platelet counts were observed in 4.9% of patients in the low-risk group, 11.5% in the indeterminate group and 69.0% in the high-risk group. Anemia rose from 14.3 to 18.4% and 44.8% across the same categories, while neutropenia increased from 3.1 to 3.4% and 10.3%, respectively.

### Aim 3: multivariable risk factor analysis

In multivariable logistic regression models adjusted for age, sex, diabetes, hypertension and dyslipidemia (Model 1; Table 5), older age was independently associated with higher odds of all three hematologic outcomes. Each additional year of age increased the odds of anemia [adjusted OR (aOR) 1.03, 95% CI 1.01–1.05], neutropenia (aOR 1.07, 95% CI 1.04–1.10) and abnormal platelet counts (aOR 1.04, 95% CI 1.03–1.06; all *p* < 0.001). Female sex was strongly associated with anemia (aOR 2.53, 95% CI 1.77–3.64) and abnormal platelets (aOR 1.77, 95% CI 1.20–2.63). Diabetes was also associated with higher odds of anemia (aOR 1.62, 95% CI 1.04–2.56) and abnormal platelets (aOR 2.10, 95% CI 1.25–3.64). In contrast, dyslipidemia showed an inverse association with anemia (aOR 0.40, 95% CI 0.26–0.60) and neutropenia (aOR 0.39, 95% CI 0.19–0.79).

**TABLE 5:** Multivariable logistic regression Model 1: demographics and comorbidities.

Predictor	Anemia		Neutropenia		Abnormal platelets	
	aOR (95% CI)	*P*-value	aOR (95% CI)	*P*-value	aOR (95% CI)	*P*-value
Age (per 1 year)	1.03 (1.01–1.05)	< 0.001	1.07 (1.04–1.10)	< 0.001	1.04 (1.03–1.06)	< 0.001
Female vs. Male	2.53 (1.77–3.64)	< 0.001	1.19 (0.62–2.31)	0.61	1.77 (1.20–2.63)	0.004
Diabetes	1.62 (1.04–2.56)	0.03	0.97 (0.44–2.20)	0.94	2.10 (1.25–3.64)	0.006
Hypertension	1.04 (0.68–1.60)	0.84	0.48 (0.22–1.04)	0.06	0.96 (0.61–1.54)	0.88
Dyslipidemia	0.40 (0.26–0.60)	< 0.001	0.39 (0.19–0.79)	0.009	0.78 (0.50–1.23)	0.28

**FIGURE 5:**
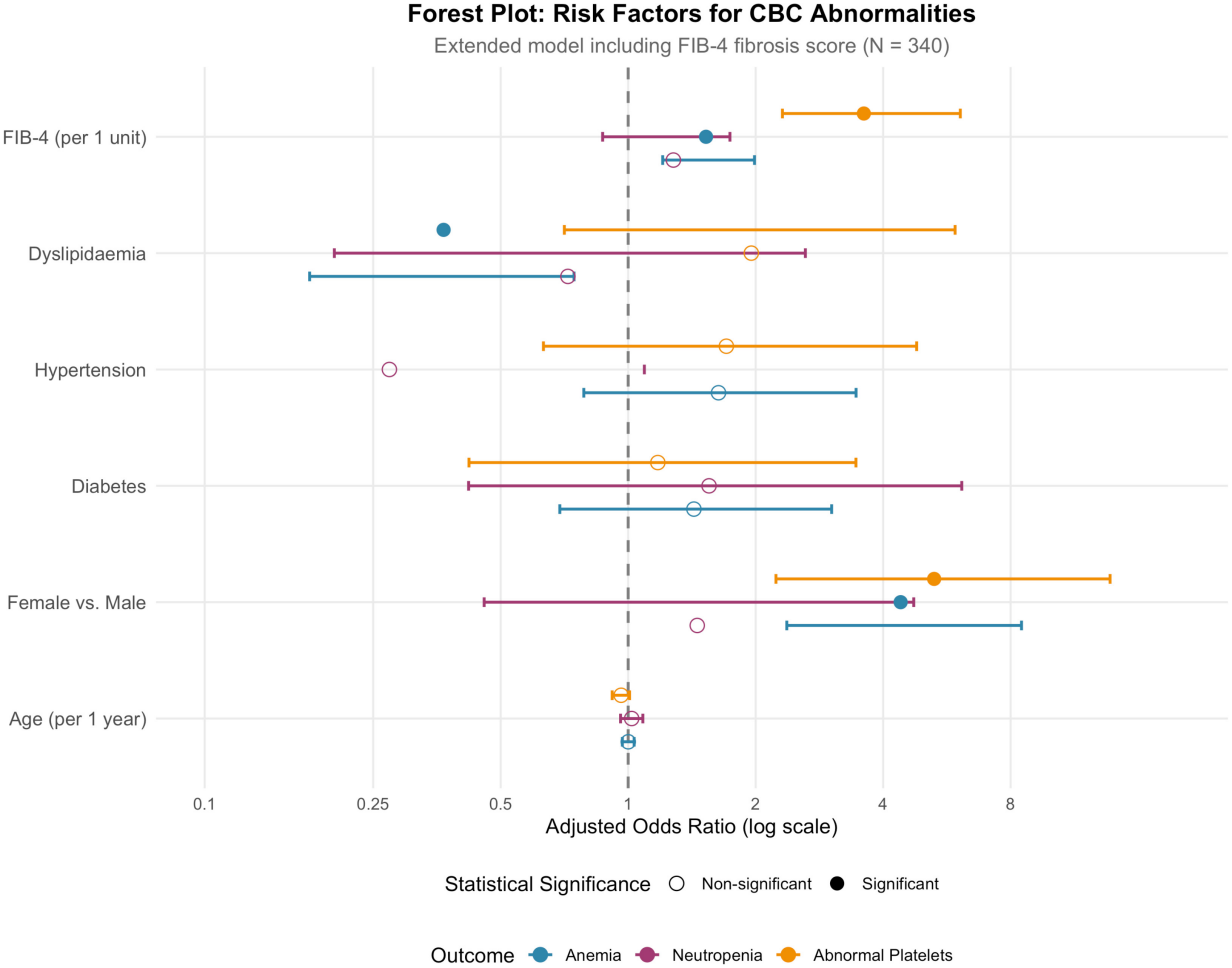
Forest plot: risk factors for CBC abnormalities (extended model with FIB-4).

When FIB-4 was added to the models (Model 2; Table 6), the strength and pattern of associations changed. Age was no longer independently associated with any of the outcomes, reflecting the incorporation of age into the FIB-4 formula. FIB-4 itself was a strong predictor of anemia (aOR 1.53 per one-unit increase, 95% CI 1.21–1.99; *p* < 0.001) and abnormal platelet counts (aOR 3.60, 95% CI 2.31–6.09; *p* < 0.001), but not neutropenia. Female sex remained strongly associated with both anemia (aOR 4.40, 95% CI 2.37–8.49) and abnormal platelets (aOR 5.28, 95% CI 2.23–13.74). These associations are summarized visually in the forest plot in Figure 5.

**TABLE 6:** Multivariable logistic regression Model 2: including FIB-4 fibrosis score.

Predictor	Anemia		Neutropenia		Abnormal Platelets	
	aOR (95% CI)	*P*-value	aOR (95% CI)	*P*-value	aOR (95% CI)	*P*-value
Age (per 1 year)	1.00 (0.97–1.03)	0.98	1.02 (0.96–1.08)	0.54	0.96 (0.92–1.01)	0.11
Female vs. Male	4.40 (2.37–8.49)	< 0.001	1.46 (0.46–4.72)	0.52	5.28 (2.23–13.74)	< 0.001
Diabetes	1.43 (0.69–3.02)	0.34	1.55 (0.42–6.13)	0.51	1.17 (0.42–3.45)	0.76
Hypertension	1.63 (0.79–3.45)	0.19	0.27 (0.06–1.09)	0.08	1.71 (0.63–4.80)	0.30
Dyslipidemia	0.37 (0.18–0.75)	0.006	0.72 (0.20–2.62)	0.61	1.95 (0.71–5.92)	0.21
FIB-4 (per 1 unit)	1.53 (1.21–1.99)	< 0.001	1.28 (0.87–1.74)	0.14	3.60 (2.31–6.09)	< 0.001

*N* = 340 with complete FIB-4 data. FIB-4 incorporates age and platelet count; associations with platelet abnormalities should be interpreted considering this mathematical coupling.

Model comparison using the Akaike information criterion (AIC) favored the extended models with FIB-4 over the base models for all three outcomes (Supplementary Table 6), although this comes at the cost of a reduced sample size (*n* = 340) due to missing liver enzyme data.

### Sensitivity analysis for dyslipidemia

Given the unexpected inverse association between dyslipidemia and some CBC abnormalities in the multivariable models, we performed a descriptive sensitivity analysis stratified by dyslipidemia status (Supplementary Table 7). NASH patients with dyslipidemia were, on average, 10 years older than those without (mean 54.8 vs. 44.6 years) and had higher mean FIB-4 scores (1.55 vs. 1.08). While the crude prevalence of anemia and neutropenia was slightly lower among patients with dyslipidemia, platelet abnormalities were more frequent (21.1% vs. 14.9%). Taken together, these findings suggest that the apparent “protective” effect of dyslipidemia in adjusted models likely reflects residual confounding, potential effects of statin therapy, or selection of patients engaged in preventive care rather than a true biological protection against cytopenias.

## Discussion

In this large, matched case–control study of adults attending primary care in Qatar, we found that hematological abnormalities were common in patients with clinically diagnosed NASH and occurred more frequently than in controls without NASH. Approximately one-third of NASH patients had at least one predefined CBC abnormality, and platelet abnormalities—particularly thrombocytopenia—showed the strongest association with NASH and with higher non-invasive fibrosis scores. Within the NASH cohort, older age, female sex and diabetes were consistently associated with anemia and platelet disturbances, whereas the relationship with dyslipidemia was more complex. Together, these findings position simple CBC parameters as clinically informative markers within the broader MASLD phenotype in a high-prevalence Middle Eastern primary care setting.

### Platelet abnormalities and fibrosis

Our data show a sevenfold higher odds of thrombocytopenia in NASH compared with matched controls, and a steep, stepwise increase in platelet abnormalities across FIB-4 fibrosis categories, with nearly 70% of patients in the high-risk FIB-4 group exhibiting abnormal platelet counts. These observations are consistent with the well-described link between thrombocytopenia and advanced chronic liver disease, where portal hypertension, hypersplenism, reduced thrombopoietin production, bone-marrow suppression and immune-mediated platelet destruction all contribute to low platelet counts (13–17). In patients with cirrhosis, the prevalence and severity of thrombocytopenia closely parallel liver disease stage and are strong predictors of adverse outcomes (15, 16).

Evidence specific to NAFLD/MASLD has been more limited. Small cohort studies have reported that a minority of non-cirrhotic NAFLD patients develop thrombocytopenia, but that platelet counts decline with increasing histological fibrosis stage (18–20). Other work has highlighted platelet indices (mean platelet volume and platelet distribution width) as potential non-invasive markers of NASH and fibrosis (8, 21). Our results extend this literature by demonstrating, in a primary-care cohort, that platelet abnormalities are not only more frequent in clinically diagnosed NASH than in controls but also strongly concentrated in those with higher FIB-4 scores. This reinforces the central role of platelet count in composite fibrosis scores such as FIB-4 and APRI and supports its use as a readily available, low-cost signal of more advanced liver disease (10, 12, 22–25).

### Anemia, neutropenia and systemic manifestations of NASH

Anemia affected nearly one quarter of NASH patients in our study, a prevalence lower than that reported in cohorts with decompensated cirrhosis but similar to estimates in compensated advanced liver disease (15, 17, 26). In chronic liver disease, anemia typically arises from a combination of gastrointestinal blood loss, nutritional deficiencies (iron, folate, vitamin B_12_), chronic inflammation, hemolysis and hypersplenism (14, 15, 17). Our cross-sectional dataset lacked detailed information on these mechanisms, including red cell indices (MCV, RDW) that would allow characterization of anemia subtypes; nevertheless, the higher FIB-4 and APRI scores observed in anemic compared with non-anemic NASH patients are compatible with a contribution from portal hypertension and more advanced liver injury. The independent association between diabetes and anemia in the base model also mirrors the broader link between metabolic dysfunction, chronic kidney disease and anemia in MASLD populations (5, 6).

Neutropenia was relatively uncommon (5.4% of NASH patients) and only weakly associated with fibrosis scores. In advanced portal hypertension, splenic sequestration can lead to leukopenia alongside thrombocytopenia (13, 14), but most of our cohort likely had earlier-stage disease, as reflected by the predominance of low or indeterminate FIB-4 scores. It is also possible that some episodes of neutropenia were transient or treatment-related (for example, due to myelotoxic medications), which we were unable to capture. Overall, our data suggest that, in a primary-care NASH population, neutropenia is a less prominent feature than anemia or thrombocytopenia.

More broadly, our findings add to the growing recognition of MASLD as a multisystem disease with extrahepatic manifestations, including cardiovascular disease, chronic kidney disease and hematological changes (5–7). The clustering of anemia and platelet abnormalities with higher non-invasive fibrosis scores supports the view that these hematologic features may act as accessible markers of more advanced systemic and hepatic involvement.

### Age, sex, and metabolic comorbidities

We found that older age and female sex were strongly associated with anemia and platelet abnormalities in the base models. Age-related declines in WBC and platelet counts, together with changes in hemoglobin, are well documented in population studies and are influenced by both biological aging and comorbidity burden (27–29). Sex differences in hematological parameters are also well established: men tend to have higher hemoglobin and hematocrit, whereas women may have slightly higher platelet counts, reflecting hormonal influences and differences in erythropoietin and iron metabolism (30, 31). Our results are consistent with these patterns and highlight that, within NASH, older women—particularly those with diabetes—represent a subgroup at higher risk of anemia and platelet abnormalities.

Diabetes was independently associated with anemia and platelet abnormalities in the base model but lost significance once FIB-4 was included, suggesting that part of its effect is mediated through more advanced liver disease or other complications. Diabetes and low platelet count are key components of several non-invasive fibrosis algorithms for NAFLD (10, 12, 24), and our findings support their continued use in risk stratification.

### Dyslipidemia and the “statin paradox”

A notable and somewhat counterintuitive finding was the inverse association between dyslipidemia and both anemia and neutropenia in the adjusted models. This finding should be regarded as hypothesis-generating and interpreted with considerable caution rather than as a definitive biological effect. The descriptive sensitivity analysis stratified by dyslipidemia (Supplementary Table 7)—now including statistical comparisons—showed that patients with dyslipidemia were on average about 10 years older and had higher mean FIB-4 scores, yet exhibited slightly lower prevalences of anemia and neutropenia and only modestly higher rates of platelet abnormalities. Patients with dyslipidemia in a primary care setting typically undergo more frequent medical monitoring, which may result in systematic differences in the completeness of laboratory testing, disease detection, and comorbidity recording. These surveillance differences, rather than a biological protective effect, may explain much of the apparent inverse association.

In particular, statins are widely prescribed for dyslipidemia and are increasingly recognized as safe in patients with MASLD (32–36). Experimental and clinical studies suggest that statins may improve hepatic steatosis, reduce inflammation and slow fibrosis progression, possibly through pleiotropic effects on endothelial function, stellate cell activation and immune modulation (33, 36, 37). Statin use has also been associated with reduced portal pressure and lower risk of decompensation in cirrhosis (33). Although we lacked direct data on statin prescriptions, it is plausible that a substantial proportion of patients labeled as having dyslipidemia were receiving statins, which could mitigate some inflammatory or fibrotic drivers of cytopenias. Alternatively, dyslipidemia may act as a proxy marker for patients more engaged with preventive care and screening, who differ systematically from those without recorded dyslipidemia. These possibilities underscore the need for future studies with detailed medication data to disentangle the direct and indirect effects of statins on hematologic parameters in MASLD.

### Comparison with non-invasive fibrosis tools

The strong association between FIB-4 and both anemia and platelet abnormalities in our extended models is expected, given that FIB-4 explicitly incorporates age, AST and platelet count in its formula (11, 22, 23, 25). To understand the conceptual impact of this mathematical coupling: a patient with thrombocytopenia (platelet count < 150 × 10^9^/L) will, all else being equal, have a higher FIB-4 value because platelet count appears in the denominator of the FIB-4 formula. Consequently, a regression model predicting platelet abnormality from FIB-4 is partially circular—the platelet component of FIB-4 explains some variance in platelet abnormality by construction rather than through independent biology. The very large OR observed for FIB-4 predicting platelet abnormality (aOR 3.60, 95% CI 2.31–6.09) should therefore be interpreted as reflecting this coupling alongside genuine biological associations. The association with anemia, which does not enter the FIB-4 formula, is less susceptible to this limitation and more likely reflects independent biological pathways. To partially address this concern, we conducted a supplementary analysis replacing FIB-4 with APRI. Since APRI incorporates AST and platelet count but excludes age, it is less mathematically coupled with age-related outcomes than FIB-4. The directionality and statistical significance of associations were preserved in APRI-based models (Supplementary Table 9), supporting the robustness of our findings.

### Characterization of patients with missing FIB-4 data

The restriction of fibrosis-related analyses to NASH patients with available AST and ALT data (approximately 38% of the cohort) raises concern about selection bias. Comparison of NASH patients with versus without available FIB-4 data (Supplementary Table 8) revealed that patients with available data were on average 1.7 years younger, and had lower rates of anemia (17.9% vs. 28.0%) and platelet abnormalities (12.1% vs. 23.8%). These data suggest that missing enzyme data was not random; patients with available measurements likely had more intensive clinical monitoring, potentially because of more symptomatic or advanced disease. The fibrosis-related estimates in Model 2 should therefore be interpreted as possibly reflecting a subset with greater disease severity and may overestimate the associations that would be observed in the full NASH cohort.

Nevertheless, even small increments in FIB-4 within the non-cirrhotic range were accompanied by clinically meaningful increases in CBC abnormality prevalence. This is consistent with prior work showing that FIB-4 and APRI have good negative predictive value for excluding advanced fibrosis in NAFLD and are recommended as first-line tools in primary care algorithms (10, 12, 22, 24). Our findings suggest that incorporating CBC abnormalities—particularly thrombocytopenia—into clinical workflows may help flag patients at highest risk in resource-limited settings.

### Strengths and limitations

Key strengths of this study include the relatively large sample of clinically diagnosed NASH patients drawn from a comprehensive primary care database, the use of matched controls from the same underlying population, and the integration of routinely collected electronic health record data with detailed laboratory measurements. This design allowed us to quantify CBC abnormalities in a real-world, primary-care MASLD population in a region with a particularly high burden of disease and metabolic risk factors (3, 4).

Several limitations should also be acknowledged. First, the cross-sectional nature of the analysis precludes causal inference and prevents assessment of temporal changes in CBC parameters or fibrosis scores. Second, NASH was identified using ICD-10 codes assigned by primary care physicians rather than histological confirmation. The ICD-10 code K75.81 is specifically designated for NASH and was supplemented with related fatty liver codes (K76.0, K76.89) in this analysis. However, the distinction between simple steatosis, NASH, and more advanced fibrotic disease is not always consistently applied in clinical practice, and some degree of misclassification is inevitable. A sensitivity analysis restricted solely to the K75.81 code was not feasible due to insufficient frequencies of the more specific code alone in this dataset. Future studies should aim for histological confirmation or standardized clinical diagnosis criteria. Third, liver disease severity was assessed using non-invasive scores rather than histology; misclassification of fibrosis stage is possible, although FIB-4 and APRI have been validated in NAFLD (10, 12, 23, 25). Fourth, we lacked information on key potential confounders, including iron, vitamin B_12_ and folate status; red cell indices (MCV, RDW) for anemia subtyping; gastrointestinal blood loss; bone-marrow disorders; and the use of medications such as statins, antiplatelet agents or myelotoxic drugs. These factors may contribute to cytopenias independent of NASH. Fifth, liver enzyme data were missing for approximately 62% of NASH patients; characterization of those with missing data (Supplementary Table 8) suggests this was not random, and fibrosis-related estimates may overrepresent patients with more advanced or closely monitored disease. Sixth, because FIB-4 includes age and platelet count, models that simultaneously include FIB-4 and platelet abnormalities should be interpreted with caution, as part of the association is mathematical rather than purely biological; a supplementary APRI-based analysis (Supplementary Table 9) supports the directionality of our findings. Seventh, hematological analyzer models were not uniformly recorded in the electronic health records, precluding analyzer-specific quality assessment. Finally, our cohort was derived from a single country and predominantly Middle Eastern population; generalizability to other ethnicities and health-care systems requires confirmation.

### Clinical and research implications

From a clinical perspective, our findings support the routine review of CBC parameters in patients with NASH or suspected MASLD in primary care. The presence of thrombocytopenia or other platelet abnormalities should prompt careful evaluation for advanced fibrosis, including calculation of FIB-4 and, where appropriate, referral for elastography or specialist assessment. Anemia in NASH warrants a structured work-up that considers both liver-related mechanisms and common comorbid conditions such as chronic kidney disease and gastrointestinal bleeding. CBC abnormalities may also help clinicians prioritize patients for more intensive monitoring or lifestyle and pharmacological interventions.

Future research should explore longitudinal trajectories of CBC parameters in MASLD and their relationship with incident cirrhosis, decompensation and cardiovascular events. Studies that integrate detailed medication data, including statin and antiplatelet use, red cell indices for anemia characterization, and imaging or histological confirmation of portal hypertension would help clarify mechanistic pathways linking MASLD, fibrosis and cytopenias. Finally, interventional trials could examine whether therapies that modify platelet activation or improve metabolic control translate into improvements in both liver outcomes and hematologic profiles.

## Conclusion

This study demonstrates that hematological abnormalities—particularly thrombocytopenia and anemia—are significantly more prevalent in patients with clinically diagnosed NASH than in matched controls and are closely linked to markers of hepatic fibrosis. Among NASH patients, nearly one-third exhibited at least one CBC abnormality, with thrombocytopenia showing a sevenfold increase compared with controls and a striking dose–response relationship across FIB-4 risk categories. Older age, female sex, and diabetes emerged as consistent risk factors for these cytopenias.

These findings carry important implications for primary care practice. The complete blood count—a universally available, inexpensive, and routinely performed test—may serve as a practical screening tool to identify NASH patients at higher risk of advanced fibrosis who warrant further evaluation with non-invasive fibrosis scores, elastography, or hepatology referral. In resource-constrained settings where access to specialized liver assessment is limited, vigilant attention to platelet counts and hemoglobin levels could help prioritize patients for targeted intervention.

Future prospective studies should examine the temporal dynamics of CBC abnormalities in MASLD, their predictive value for clinical endpoints such as cirrhosis and liver-related mortality, and the potential impact of therapeutic interventions—including lifestyle modification and pharmacotherapy—on both liver and hematological outcomes. Such research will be essential to establish whether routine CBC monitoring can be formally integrated into MASLD care pathways and risk stratification algorithms.

## Data Availability

The raw data supporting the conclusions of this article will be made available by the authors, without undue reservation.
