# Supplementary material for: Hematological abnormalities in clinically diagnosed non-alcoholic steatohepatitis: prevalence, clinical correlates, and fibrosis risk in a case–control study from Qatar
Source: Front Med (Lausanne). 2026 May 8;13:1773499. doi: 10.3389/fmed.2026.1773499 (PMC13194006; doi:10.3389/fmed.2026.1773499)
Supplement: Supplementary file 1 [file Data_Sheet_1.DOCX]

# Supplementary Materials

## Supplementary Tables

Table 1: Data availability for key variables among NASH patients.

| **Variable** | **Available, n/N (%)** |
| --- | --- |
| Haemoglobin | 765/894 (85.6%) |
| WBC | 765/894 (85.6%) |
| Platelets | 765/894 (85.6%) |
| AST | 341/894 (38.1%) |
| ALT | 346/894 (38.7%) |
| FIB-4 | 340/894 (38.0%) |

Table 2: Characteristics of NASH patients by anemia status.

| **Characteristic** | **No Anemia** | **Anemia** | **P-value** |
| --- | --- | --- | --- |
| N | 585 | 180 | — |
| Age, mean (SD) | 49.2 (12.7) | 54.2 (15.3) | <0.001 |
| Female, n (%) | 225 (38.5) | 116 (64.4) | <0.001 |
| Diabetes, n (%) | 347 (59.3) | 133 (73.9) | <0.001 |
| Hypertension, n (%) | 292 (49.9) | 105 (58.3) | 0.059 |
| Dyslipidaemia, n (%) | 361 (61.7) | 95 (52.8) | 0.040 |
| FIB-4, mean (SD) | 1.21 (0.90) | 2.08 (2.17) | 0.003 |
| APRI, mean (SD) | 0.48 (0.32) | 0.65 (0.63) | 0.040 |

Table 3: Characteristics of NASH patients by platelet abnormality status.

| **Characteristic** | **Normal Platelets** | **Abnormal Platelets** | **P-value** |
| --- | --- | --- | --- |
| N | 623 | 142 | — |
| Age, mean (SD) | 48.7 (13.0) | 57.8 (13.4) | <0.001 |
| Female, n (%) | 254 (40.8) | 87 (61.3) | <0.001 |
| Diabetes, n (%) | 362 (58.1) | 118 (83.1) | <0.001 |
| Hypertension, n (%) | 303 (48.6) | 94 (66.2) | <0.001 |
| Dyslipidaemia, n (%) | 360 (57.8) | 96 (67.6) | 0.040 |
| FIB-4, mean (SD) | 1.12 (0.64) | 3.18 (2.59) | <0.001 |
| APRI, mean (SD) | 0.45 (0.31) | 0.90 (0.67) | <0.001 |

Table 4: Model comparison: Base vs. Extended (with FIB-4).

| **Outcome** | **N (Base)** | **AIC (Base)** | **N (Extended)** | **AIC (Extended)** | **ΔAIC** | **Preferred Model** |
| --- | --- | --- | --- | --- | --- | --- |
| Anemia | 765 | 775.5 | 340 | 283.3 | -492.2 | Extended |
| Neutropenia | 765 | 307.4 | 340 | 117.3 | -190.1 | Extended |
| Abnormal Platelets | 765 | 673.2 | 340 | 177.2 | -496.0 | Extended |
| N = 340 with complete FIB-4 data. Lower AIC indicates better model fit. Note that models are estimated on different sample sizes due to missing FIB-4 data. | | | | | | |

Table 5: CBC abnormalities and clinical characteristics by dyslipidaemia status.

| **Dyslipidaemia** | **N** | **Mean Age (SD)** | **Mean FIB-4 (SD)** | **Anemia (%)** | **Neutropenia (%)** | **Abnormal Platelets (%)** |
| --- | --- | --- | --- | --- | --- | --- |
| No | 393 | 44.6 (12.9) | 1.08 (1.09) | 27.5 | 6.8 | 14.9 |
| Yes | 501 | 54.8 (11.7) | 1.55 (1.34) | 20.8 | 4.4 | 21.1 |
| **P-value** | **—** | **<0.001** | **<0.001** | **0.040** | **0.197** | **0.040** |
| P-values from independent t-test (continuous) or chi-square test (categorical). | | | | | | |

Table 6: Characteristics of NASH patients with versus without available FIB-4 data.

| **Characteristic** | **FIB-4 Missing** | **FIB-4 Available** | **P-value** |
| --- | --- | --- | --- |
| N | 554 | 340 | — |
| Age, mean (SD) | 51.0 (13.6) | 49.3 (12.6) | 0.064 |
| Female, n (%) | 242 (43.7) | 140 (41.2) | 0.506 |
| Diabetes, n (%) | 360 (65.0) | 196 (57.6) | 0.034 |
| Hypertension, n (%) | 299 (54.0) | 166 (48.8) | 0.154 |
| Dyslipidaemia, n (%) | 294 (53.1) | 207 (60.9) | 0.027 |
| Anemia, n (%) | 119 (28.0) | 61 (17.9) | 0.002 |
| Abnormal Platelets, n (%) | 101 (23.8) | 41 (12.1) | <0.001 |
| Comparisons by t-test (age) or chi-square (categorical variables). | | | |

Table 7: Sensitivity analysis: Multivariable logistic regression with APRI replacing FIB-4.

| **Predictor** | **Anemia: aOR (95% CI)** | **Neutropenia: aOR (95% CI)** | **Abnormal Platelets: aOR (95% CI)** | **Anemia: P-value** | **Neutropenia: P-value** | **Abnormal Platelets: P-value** |
| --- | --- | --- | --- | --- | --- | --- |
| Age (per 1 year) | 1.02 (0.99–1.05) | 1.04 (0.98–1.10) | 1.03 (0.99–1.07) | 0.170 | 0.155 | 0.107 |
| Female vs. Male | 4.32 (2.35–8.24) | 1.55 (0.50–4.98) | 3.90 (1.82–8.82) | <0.001 | 0.445 | <0.001 |
| Diabetes | 1.32 (0.64–2.76) | 1.41 (0.40–5.35) | 1.00 (0.40–2.60) | 0.449 | 0.597 | 0.995 |
| Hypertension | 1.55 (0.76–3.24) | 0.26 (0.06–1.04) | 1.47 (0.60–3.71) | 0.232 | 0.066 | 0.410 |
| Dyslipidaemia | 0.36 (0.18–0.73) | 0.69 (0.20–2.41) | 1.53 (0.62–4.08) | 0.005 | 0.547 | 0.376 |
| APRI (per 1 unit) | 2.34 (1.23–4.54) | 0.67 (0.10–2.57) | 6.30 (3.01–14.11) | 0.010 | 0.618 | <0.001 |
| N = 340 with complete APRI data. APRI = AST/platelet ratio index; excludes age from the score formula, reducing mathematical coupling compared with FIB-4. | | | | | | |
